# Characterization of Aloe Barbadensis Miller leaves as a potential electrical energy source with optimum experimental setup conditions

**DOI:** 10.1371/journal.pone.0218758

**Published:** 2019-06-25

**Authors:** Peng Lean Chong, Ajay Kumar Singh, Swee Leong Kok

**Affiliations:** 1 Centre for Communication System and IC Design (CSID), Faculty of Engineering and Technology, Multimedia University, Melaka, Malaysia; 2 Centre for Telecommunication Research & Innovation (CeTRI), Fakulti Kejuruteraan Elektronik dan Kejuruteraan Komputer (FKEKK), Universiti Teknikal Malaysia Melaka (UTeM), Melaka, Malaysia; Universiti Malaysia Pahang, MALAYSIA

## Abstract

Electrical energy can be harvested from the living plants as a new potential renewable energy source. Characterization of the electrical signal is needed to enable an optimum energy harvesting setup condition. In the present paper, an investigation is conducted to analyze the characteristic of Aloe Barbadensis Miller (Aloe Vera) leaves in terms of electrical energy generation under specific experimental setups. The experimental results show that 1111.55uW electrical power can be harvested from the Aloe Vera with 24 pairs of electrodes and this energy is capable to be stored in a capacitor. This energy has a high potential to be used to power up a low power consumption device.

## Introduction

Advancement of technology in the 21^st^ century has created a series of low power consumption and smaller size consumer electronics. This phenomenon had opened up the opportunity for the development of energy harvesting technique from low power energy sources such as from the vibration via piezoelectric materials, bioenergy from organic compounds via microbial fuel cell, radio frequency (RF) signal via RF power harvester, thermal energy via thermo-electric generator (TEG) and light energy via solar photovoltaic cell. These technologies had been well known and used to harvest micro-energy [1–6]. Hence, apart from these existing technologies, this research would like to introduce the usage of living plants as another new renewable energy source to harvest micro-energy. Certain plants can produce a continuous small amount of electrical power at both day and night, unlike solar power, which is only functional in the presence of light. This new source of energy from plants is renewable, pollution free and sustainable as long as the plant is alive. Plants are sensitive to light due to its photoreceptors, which can be categorized as phytochromes, blue/UV-A and UV-B photoreceptors [7]. The plant uses light to differentiate day and night via photoperiodism and to enable the generation of energy via photosynthesis.

Photosynthesis is a process used by plants to synthesize carbohydrate molecules from carbon dioxide and water via the usage of light energy, normally from the sun. This process will cause the transport of electrons inside the plants, which creates a potential difference between the leaves and roots under exposure of light. This phenomenon is triggered on the plant by the periodic changes of light and darkness from the light source. With such condition, a plant can generate a potential difference as much as 50mV [8–9]. Respiration in plants, on the other hand, is a reversed process of photosynthesis. It is a process of transforming the carbohydrate molecules from photosynthesis into energy for the plants. Both chemical processes induce the flow of electrons. However, the rate of photosynthesis and respiration are influenced by other environmental factors such as water, the concentration of oxygen and carbon dioxide in the air and nutrient supply available in the soil [10].

When a plant is subjected to external stimuli other than light such as mechanical stress from wounding the plant [11–13], temperature variance [14], and watering disparity [15–17], the intercellular process within the plant will produce an electric potential signal in response to these external stimuli. These responses are due to the physiological activities of plants [18–19] in the cellular cell at the microscopic level. The electric potential difference generated in the response of the physiological activities to the external stimuli is measured at most at tens of millivolts [20]. However, electrical conduction will differ from plants to plants [21–22]. As plants constitute of complex conductive and insulated elements, these will affect the electron flow ability among different species of plants. The most promising type of plants, which can generate a higher amount of electron, is the succulent family of plants [23]. Succulent plants are water-retaining plants, which can store water in their leaves, stems, and roots in order to survive in a dry environment. Hence, the conductivity of the plants is enhanced with its relatively abundant of water in its bodies. Previous research had been conducted on several different types of trees covering the non-succulent trees and succulent trees. The species of the plants covered are Alstonia scholaris (Pulai tree) and Musa acuminata (Banana tree) for non-succulent plants as well as Aloe barbadensis Miller (Aloe Vera) for succulent plant [24]. It is verified that the succulent plant produces much higher voltage compare to non-succulent plant.

Moreover, the mechanism uses to harvest electrical energy from plants will also affect the amount of energy collected from them. By embedding electrodes into the plants, an electrochemistry process happens where it converts the chemical energy to electrical energy via an oxidization-reduction reaction [25–26]. The oxidization process, which happens at the anode electrode and reduction process, which happens at the cathode electrode, causes the electron to flow from anode to cathode to produce electricity. With this method, the plant’s organic matter is functioning as an electrolyte between the two electrodes. This system is termed as Plant Based Cell (PBC) in this research. It provides a direct method to harvest DC current and voltage from the plants, which can be potentially used to power up ultra-low power devices. However, there are several aspects to be considered in the setup of the electrochemistry process that will influence the magnitude of electricity generated. First, the different types of material used as the electrode pairs. Second, the number of electrode pairs and third, the connection method between the electrodes.

The objective of the present paper is to investigate the characteristics of the Aloe Vera plant as a potential energy source and to determine its optimum setup to harvest a higher amount of energy from the plant. The whole paper is organized as; section II, which explains the various experimental setups to investigate the conditions to harvest maximum energy from the plant, section III, which discusses the experimental findings in detail, and section IV, which concludes the paper.

## Materials and methods

Experiments are performed under various conditions to study the different aspects that influence the output of energy harvested from the Aloe plant. There are around 250 species of Aloe plants. The species of the Aloe plant selected for this experiment is the Aloe Barbadensis Miller, which is more commonly known as the Aloe Vera. Each of the Aloe Vera selected is approximately 3 years old and a fully-grown plant. The size of the selected plant is approximately 50 to 60 cm in height and 50 to 70 cm in diameter from one tip of the leaf to another tip of the leaf. Each of the succulent leaves of the Aloe Vera is about 35 to 40 cm in length, with a maximum width of 6 to 7 cm and with 2 to 2.5 cm thickness. All the experiment setups are performed in an indoor laboratory environment. The room temperature is kept at 25 to 26 degrees Celsius and the indoor relative humidity percentage is kept at 56% to 61% by the indoor air conditioning system at the same time measured by a DHT 11 temperature and humidity sensor. The Aloe Vera plants are located next to a closed transparent window and subjected to the light intensity variation from the outdoor sunlight, which varies from day to night. All the voltage and current are measured by a high precision Extech EX540 multi-meter with data logging and wireless PC interface capability with an accuracy of ±0.06% for voltage measurement range from 0.01mV to 1000V and the current measurement range from 0.01μA to 20A. All the electrode pairs used in the experiments are cleaned with sandpaper and alcohol to remove contaminants before immersing them to the leaves of the Aloe Vera. The laboratory protocol to carry out the experiments setups below is available at dx.doi.org/10.17504/protocols.io.2yngfve. The experiment setups are performed in the laboratory of Centre for Communication System and IC Design (CSID), Faculty of Engineering and Technology, Multimedia University, Melaka, Malaysia. All the materials and equipment used in this study are obtained from this academic institution. The experimental setups are:

### Setup to investigate the types of electrodes

**Fig 1:**
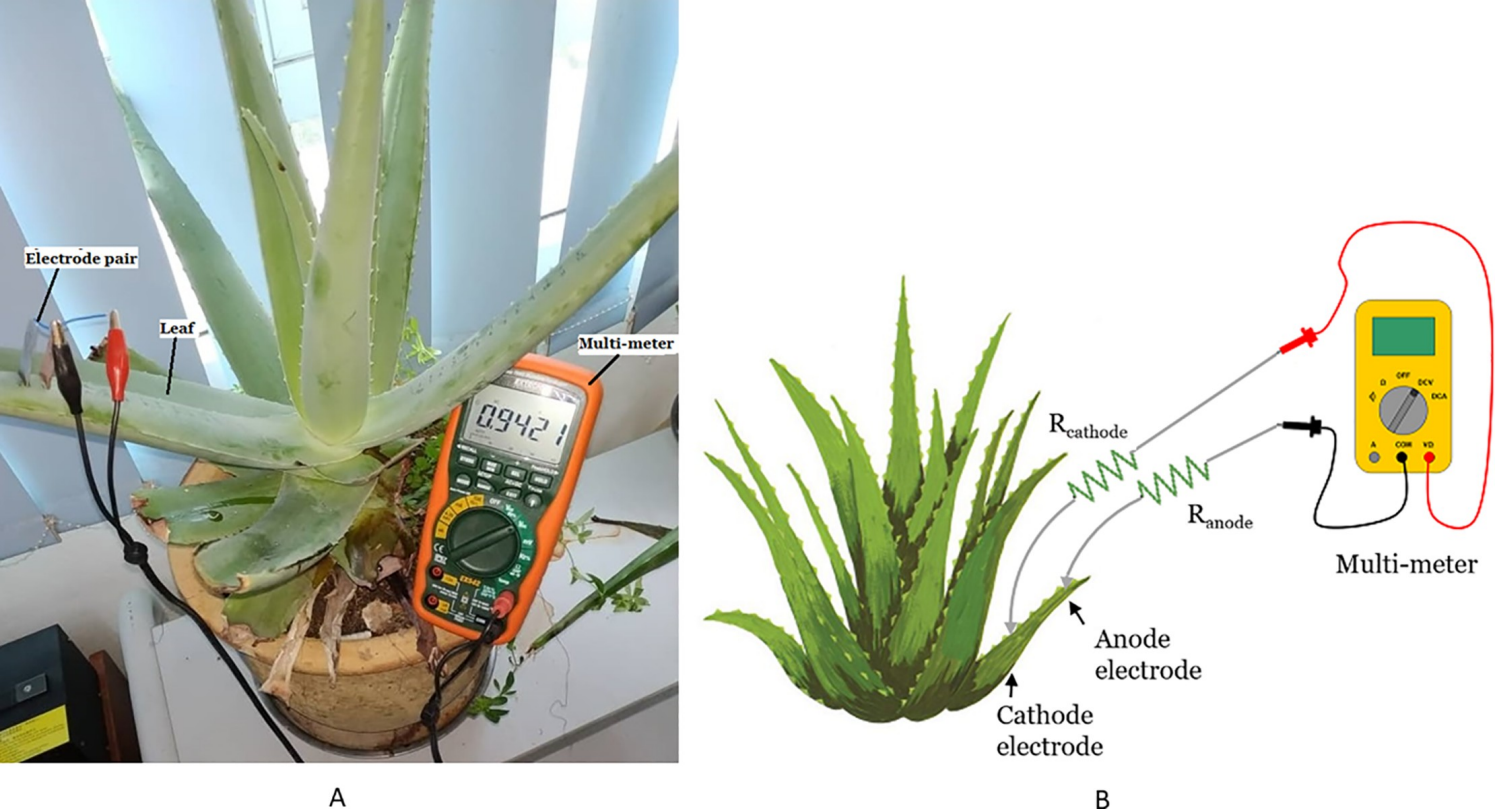
Experiment setup for harvesting energy from Aloe Vera with different electrode combinations. Example of setup arrangement (A) and simple schematic of the setup (B).

The experiment, as shown in Fig 1 is performed to identify the best pair of anode and cathode electrodes to harvest maximum voltage and current from the plant.

A pair of electrodes from different material is embedded into the leaf of an Aloe Vera plant to implement a PBC system. Copper, zinc, aluminum and nickel electrodes are chosen due to cost-effectiveness and ease of availability. The constant variables cover the size of electrode, which is fixed at 2.5 cm length, 2 cm width, and 1mm thickness. The distance between the electrodes is 1 cm and the depth of electrode penetration into the leaf is set at 1.5 cm while the light intensity is maintained. The whole experiment is performed without connecting any load.

### Setup to investigate the effect of distance between electrodes

**Fig 2:**
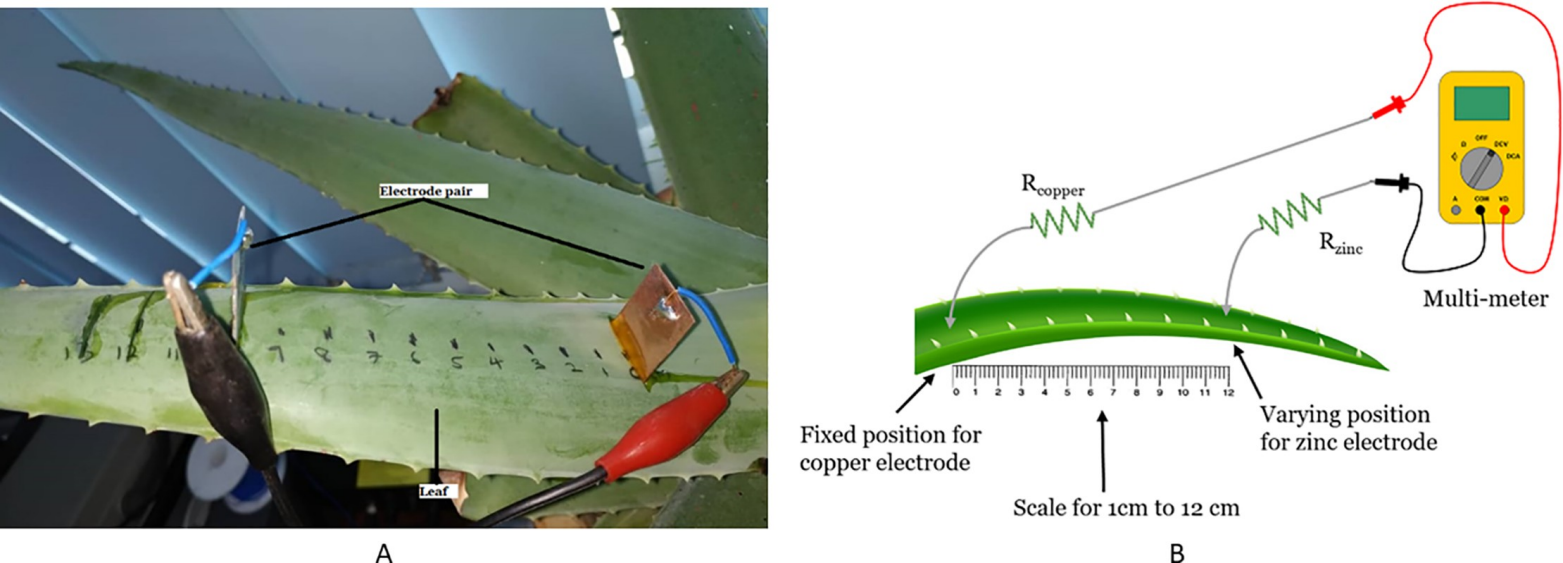
Experiment setup for variation of distance between electrodes. Example of setup arrangement (A) and simple schematic of the setup (B).

The experiment aims to identify the optimum distance between cathode and anode electrodes to harvest maximum voltage and current from the plant. The experimental set up is shown in Fig 2. The electrode pair is chosen to be copper as the cathode and zinc as the anode. The copper electrode is immersed in a fixed position at the Aloe Vera leaf located near the stem whereas the zinc electrode varies its distance from the copper electrode throughout the leaf until its tip. The distance varies in an increment of 1 cm along the leaf covering from 1–12 cm length. The size and depth of electrode penetration remain constant. The high precision multi-meter is used to measure the voltage and current.

### Setup to investigate the effect of the different location to embed electrodes on Aloe Vera

**Fig 3:**
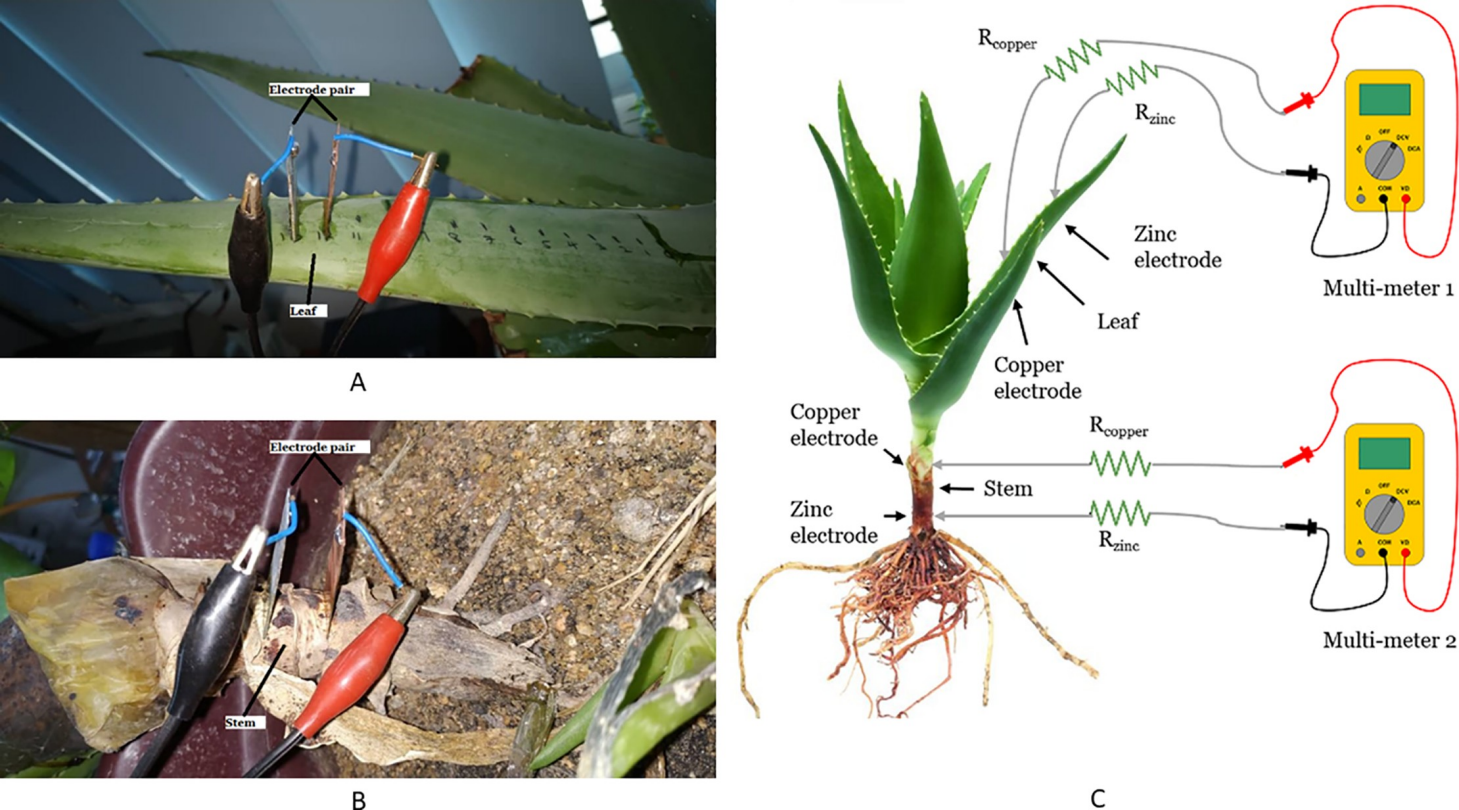
Experiment setup for embedding electrodes in leaf and stem of Aloe Vera. Setup for embedding electrodes in leaf (A), setup for embedding electrodes in stem (B) and simple schematic for both setups (C).

The experiment focuses on identifying the voltage harvested from different parts of an Aloe Vera plant, which is the stem and the leaf. An electrode pair is embedded into the leaf and the stem of the Aloe Vera plant to compare the voltage difference, which can be harvested. The size and depth of electrode penetration and measuring method remain the same as in previous experiments. The selected electrode pairs are copper and zinc. The distance between the electrodes is 1 cm. Fig 3 show the experimental setup.

### Setup to investigate the effect of the number of electrodes used

**Fig 4:**
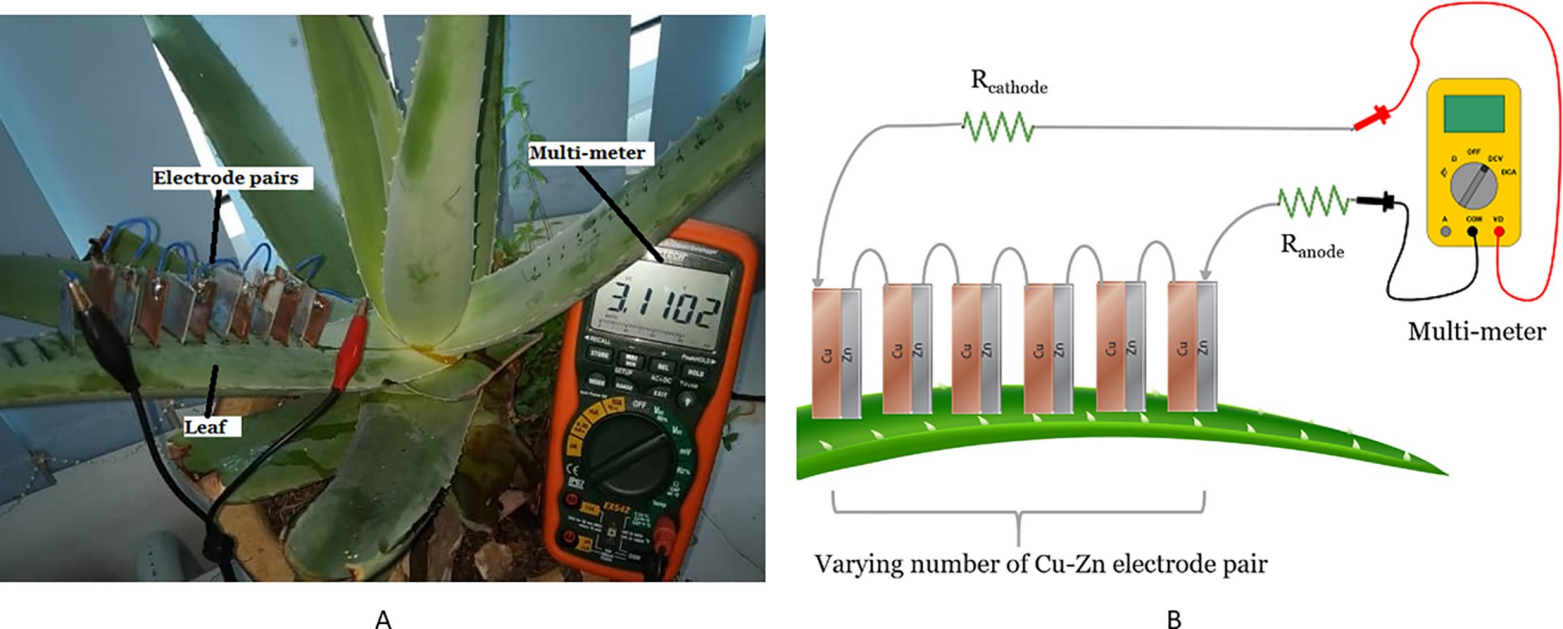
Experiment setup for a varying number of electrodes in a leaf. Example of setup arrangement (A) and simple schematic of the setup (B).

This experiment, as shown in Fig 4, aims to study the effect of the number of electrode pairs connected in series in a single Aloe Vera leaf towards the magnitude of voltage and current harvested from the plant. Copper and zinc electrode pairs are immersed in an increasing number on the leaf and the output voltage and current are measured with a multi-meter. The number of electrode pairs used ranges from 1 to 6 pairs. The distance between each electrode is maintained at 1cm. The electrode size, depth of electrode penetration, and the light intensity remain the same as in the previous experiments.

### Setup to investigate the effect of series and parallel connection among Aloe Vera leaves

**Fig 5:**
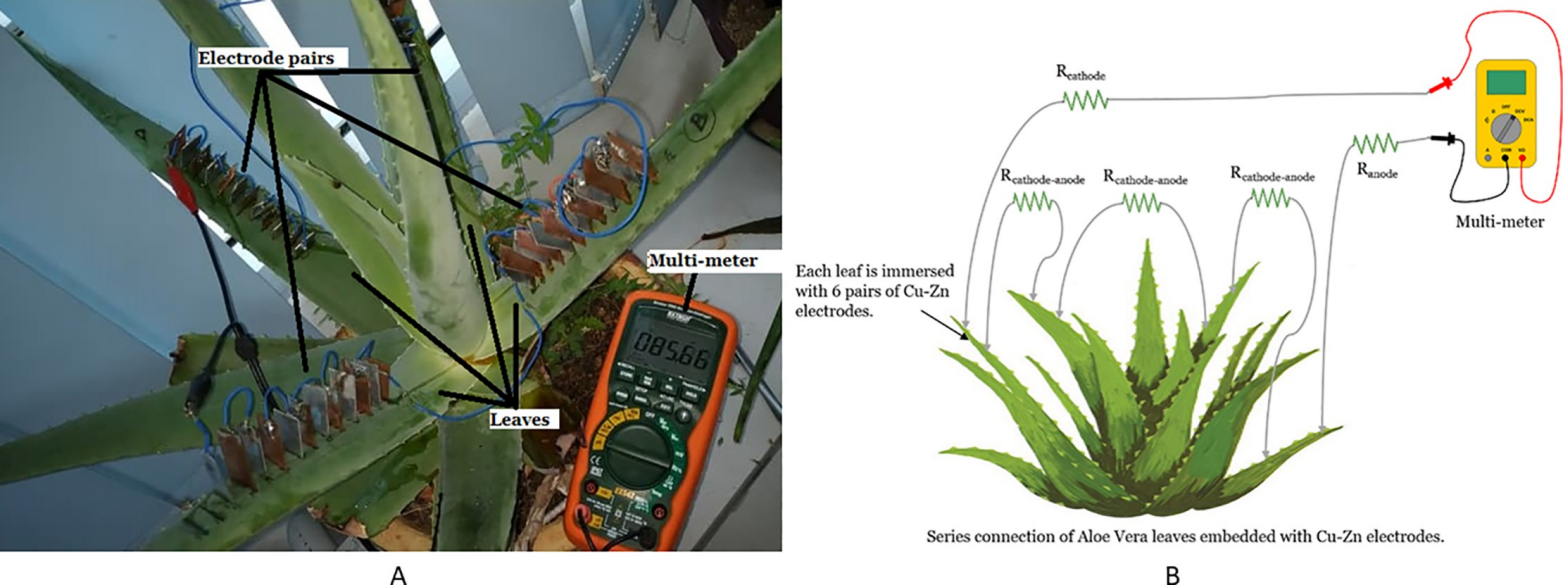
Experiment setup for series connection between leaves. Example of setup arrangement (A) and simple schematic of the setup (B).

**Fig 6:**
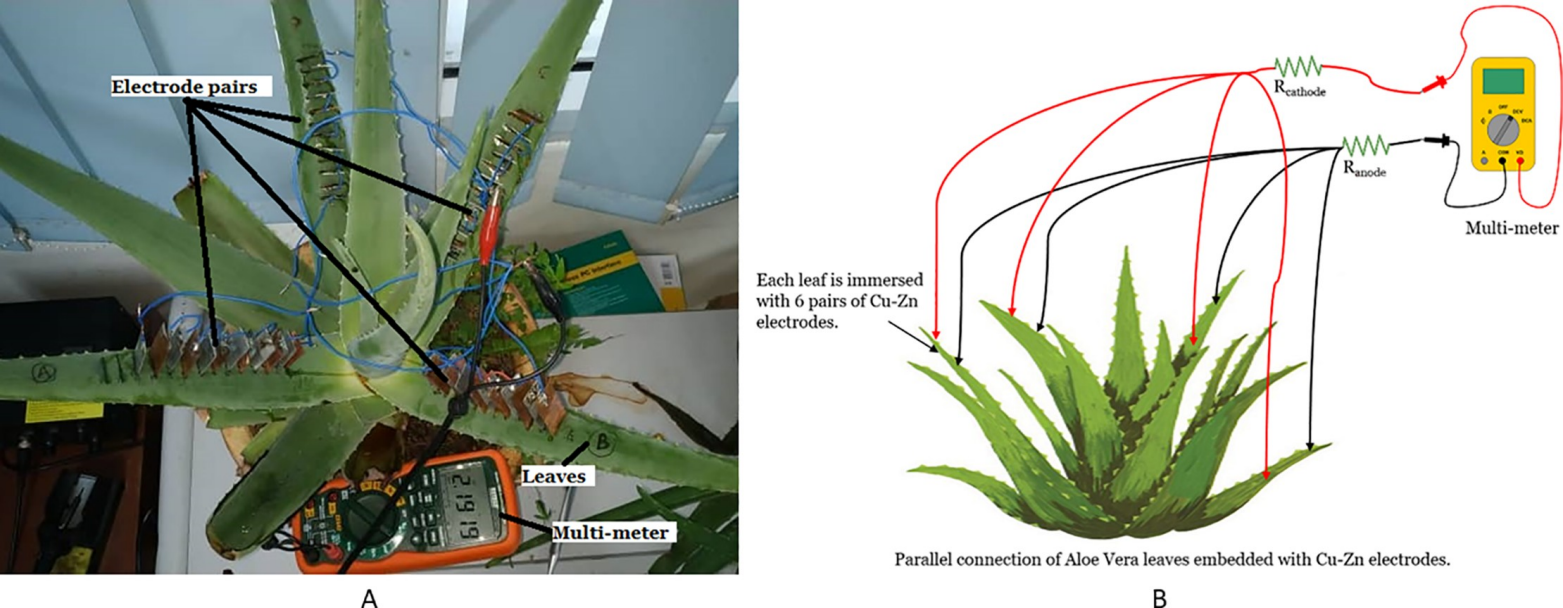
Experiment setup for parallel connection between leaves. Example of setup arrangement (A) and simple schematic of the setup (B).

This experiment investigates the effect of series and parallel connections between the Aloe Vera leaves on the magnitude of voltage and current harvested from the plant under no load condition. Four leaves were connected with electrodes. Each leaf accommodates six pairs of electrodes. These four leaves are connected in series as shown in Fig 5 as well as in parallel as shown in Fig 6 respectively. Other constant variables remain the same as in the previous experiment.

### Setup to investigate the effect on the variation of series connection among Aloe Vera leaves

**Fig 7:**
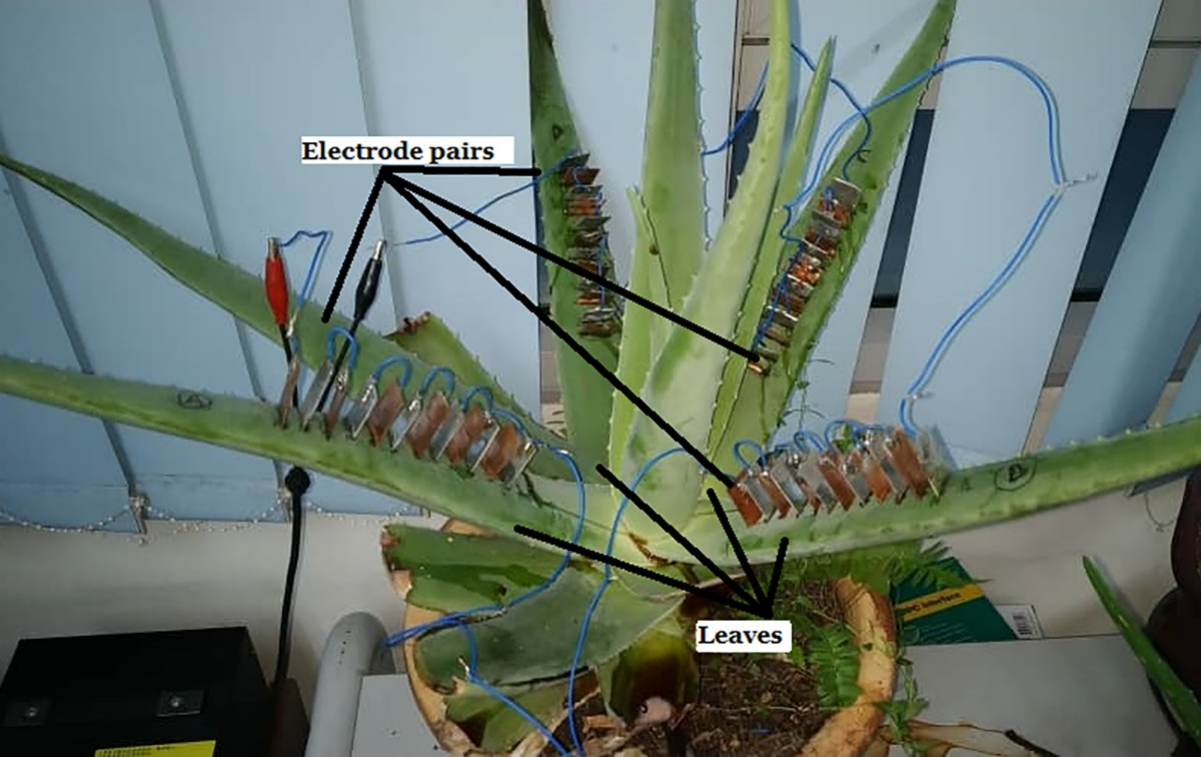
Experiment setup for series connection among leaves which passing through the stem of the Aloe Vera.

This experiment investigates the effect on the variation of series connection among the leaves towards the harvested voltage and current from the plant. Three series connection setups are investigated. It covers:

(i) the series connection among leaves which passing through the stem of the Aloe Vera as shown in Fig 7.

**Fig 8:**
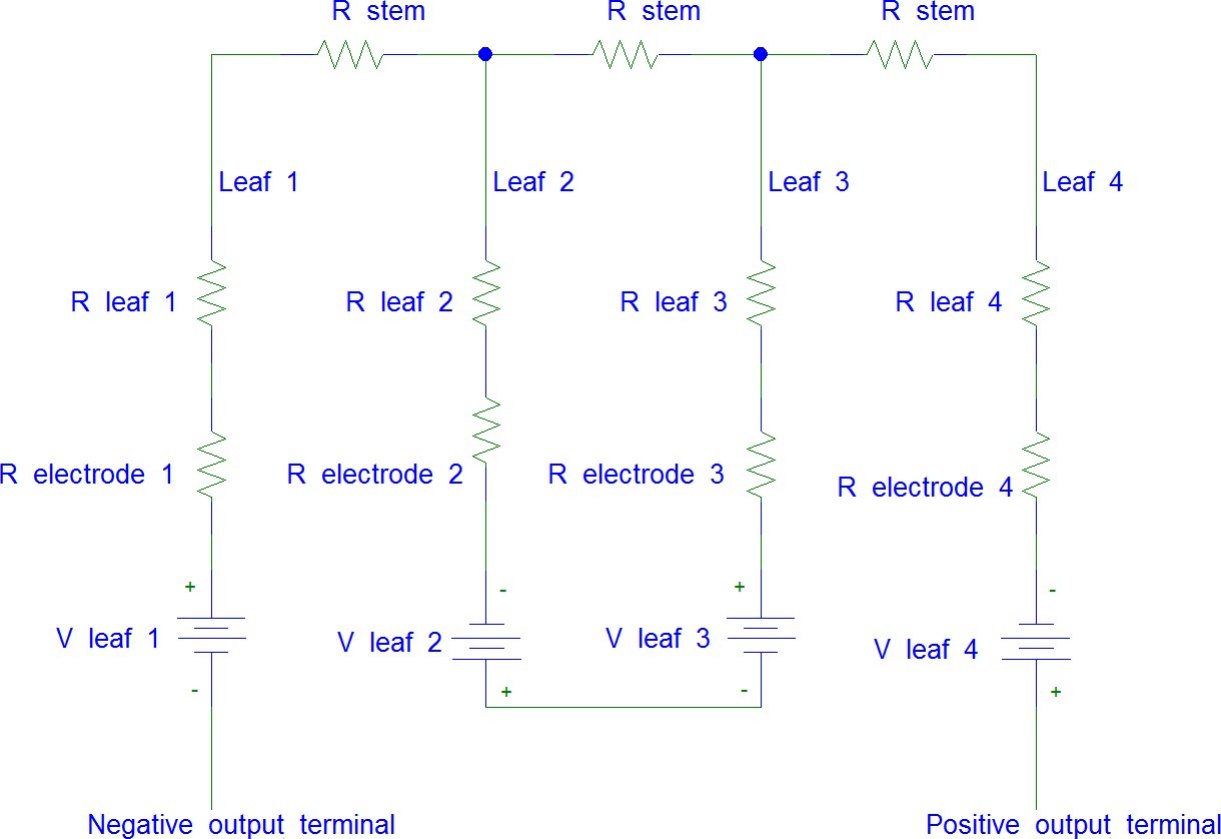
The equivalent circuit for series connection among leaves which passing through the stem of the Aloe Vera.

**Fig 9:**
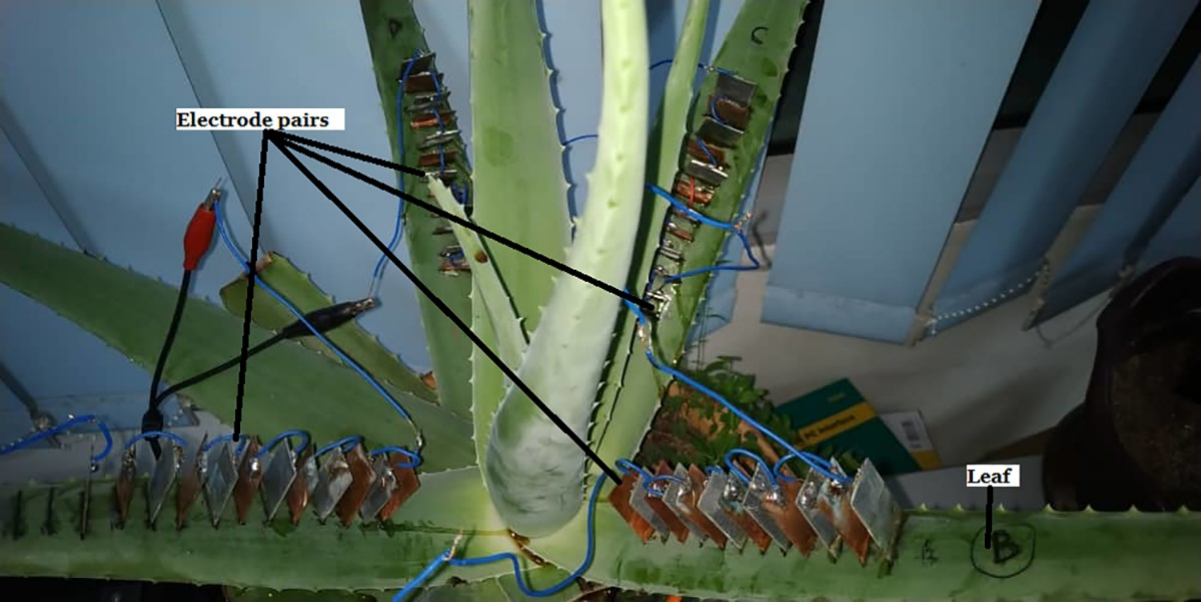
Experiment setup for series connection among leaves in which the connection by-passes the stem of the Aloe Vera.

In this setup, leaf 1 and leaf 2 are connected in series by passing through the stem, leaf 2 and leaf 3 are connected in parallel and leaf 3 and 4 are again connected in series passing through the stem. The equivalent circuit for this connection is shown in Fig 8. The polarity of the cathode and anode between each leaf are alternated consecutively. R_*leaf*_ indicates the resistance in the leaf, R_*stem*_ indicates the resistance of the stem, R_*electrode*_ shows the resistance of the electrode pairs and V_*leaf*_ refers to the voltage produced by 6 pairs of electrodes in series from a single leaf.

(ii) the series connection among the leaves in which the connection by-passes the stem of Aloe Vera as shown in Fig 9.

**Fig 10:**
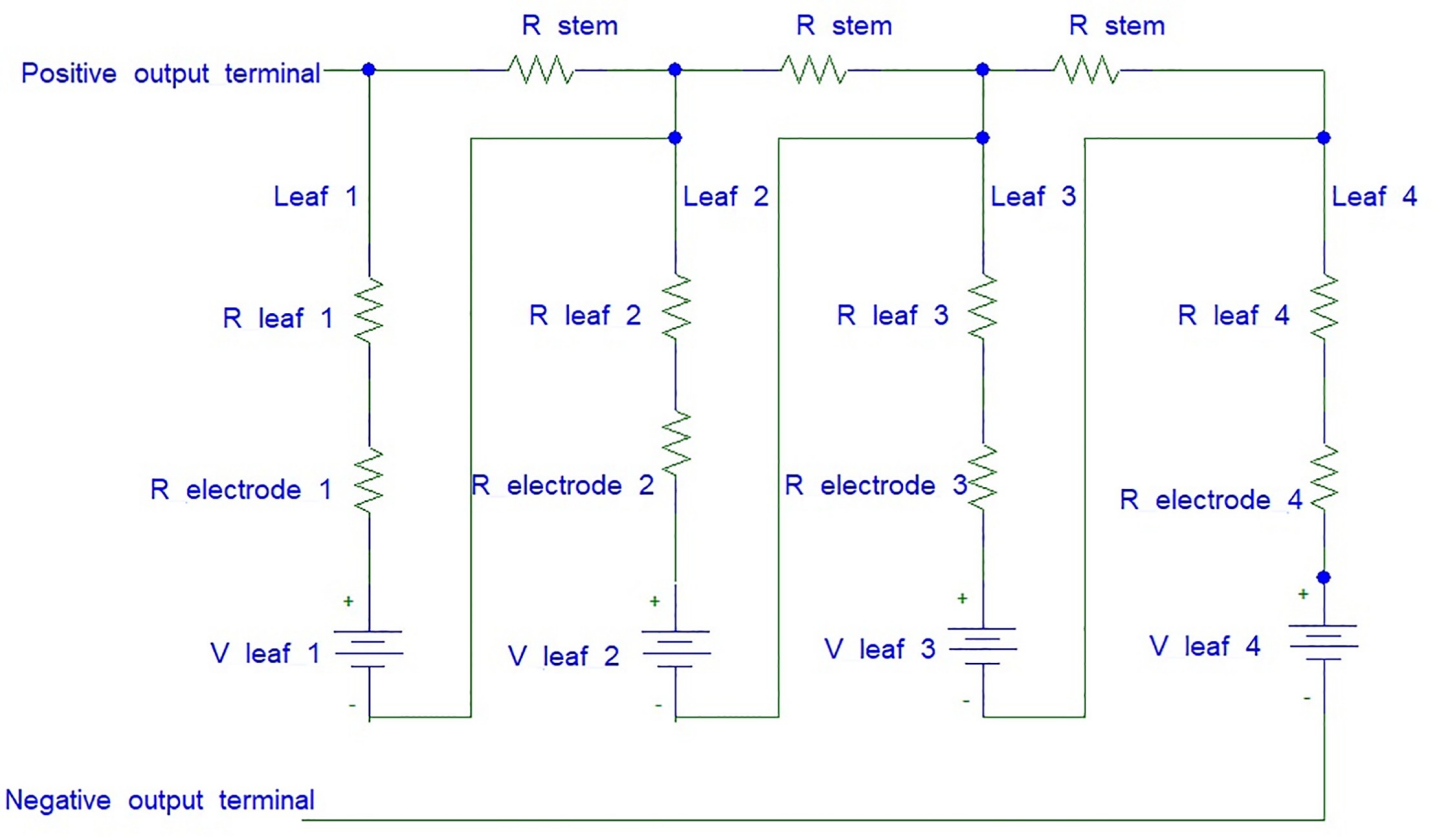
The equivalent circuit for series connection between leaves which by-pass the stem of the Aloe Vera.

**Fig 11:**
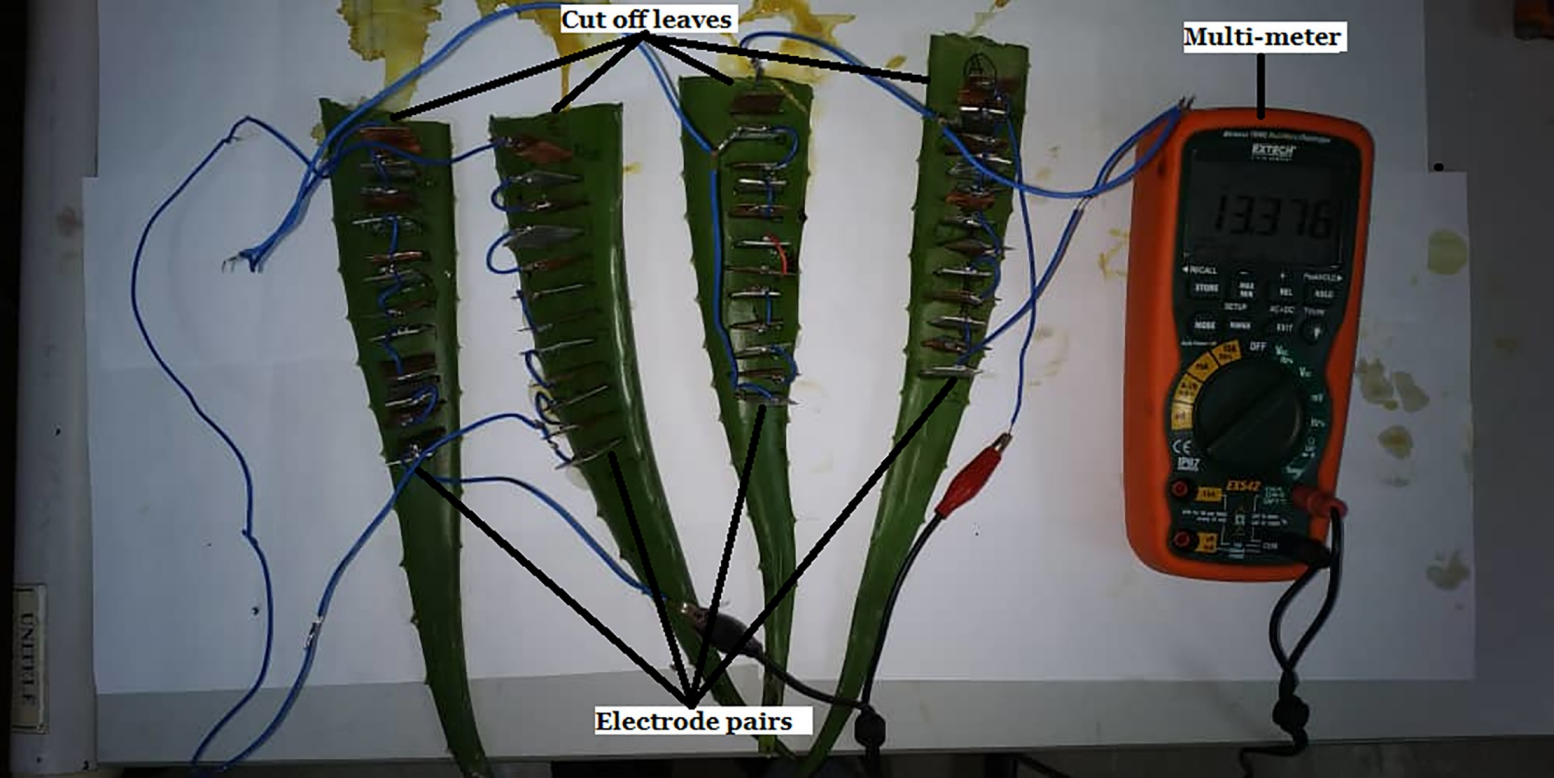
The equivalent circuit for series connection between cut off leaves.

In this setup, all the cathode electrodes are placed facing the stem and all the anode electrodes are placed facing the tip of the leaves. Hence, each electrode pairs have the same polarity configuration. The anode of the last electrode pair in each leaf is connected by wire to the cathode of the first electrode pair of another leaf to enable by-passing of the stem in the connection. The equivalent circuit for this experiment is shown in Fig 10. The notation for the circuit remains the same as in the previous experiment.

(iii) the series connection among cut off leaves which are disconnected from the stem of the Aloe Vera in Fig 11.

**Fig 12:**
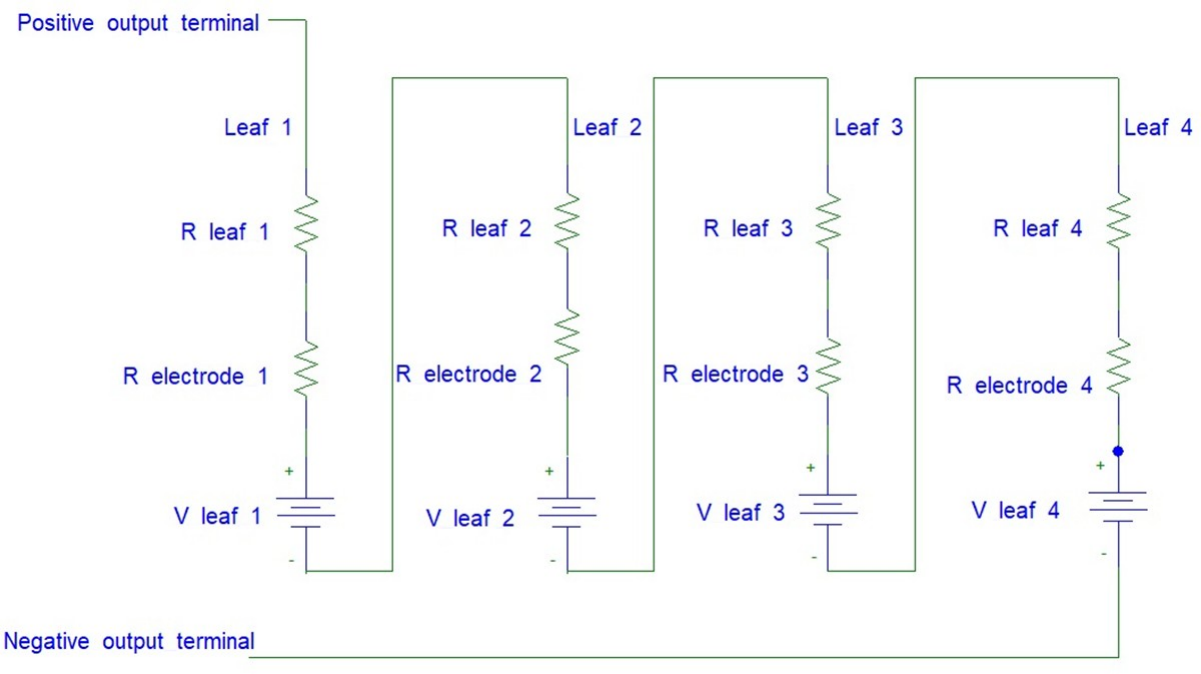
The equivalent circuit for series connection between cut off leaves.

This setup uses four cut off leaves from the Aloe Vera plant. Each leaf is connected in series as shown in the equivalent circuit of Fig 12. This setup excludes the stem from the series connection. Thus, the result can be used to compare with the previous two setups to determine the effect of the stem towards the series connection.

### Setup to investigate the effect on charging capability of the plant towards different capacitors

**Fig 13:**
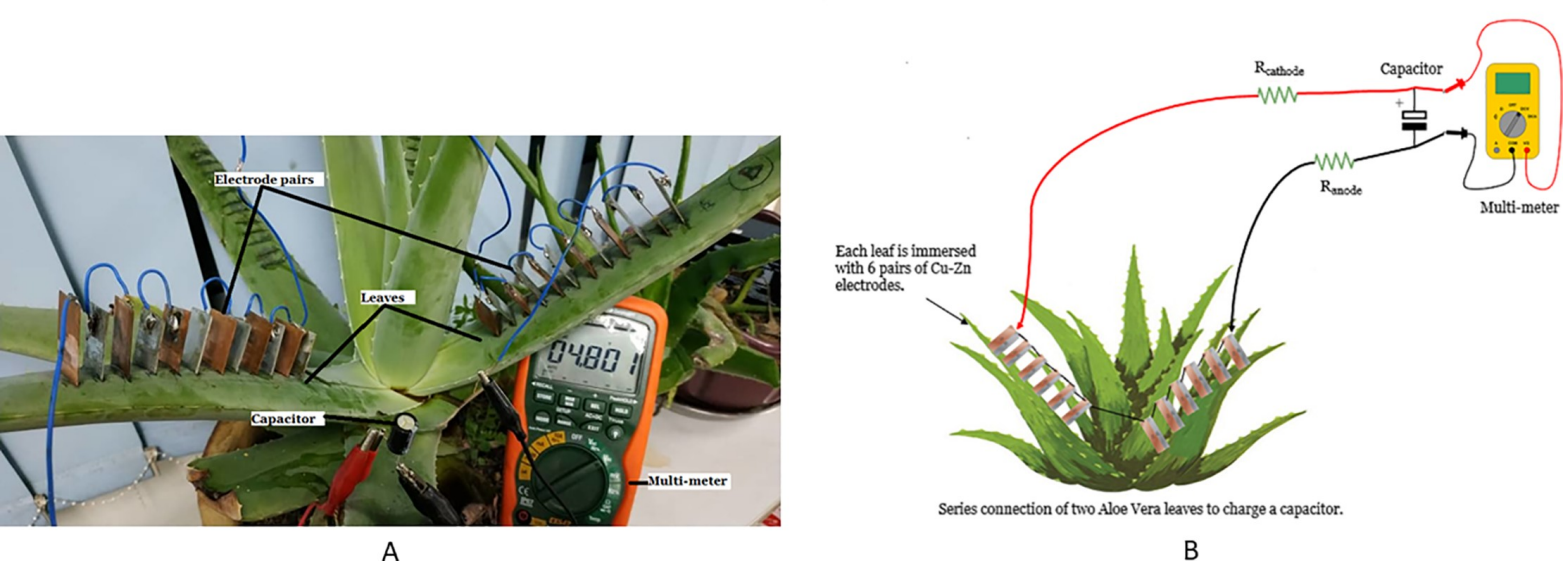
Experiment setup for charging a capacitor using the PBC system. Example of setup arrangement (A) and simple schematic of the setup (B).

This experiment investigates the feasibility of the PBC system to charge different capacitors from a wide range of capacitance value as given in Table 1. The experiment setup is shown in Fig 13. Each capacitor has been charged individually for a duration of 600 seconds. The additional constant variables are the usage of two Aloe Vera leaves connected in series, which is passing through the stem, and each leaf is embedded with 6 pairs of electrodes.

**Table 1:** Types of capacitors selected.

Capacitance	Voltage
10uF	16V
22uF	16V
47uF	16V
100uF	16V
470uF	25V
1000uF	16V
4700uF	16V

### Setup to investigate the characteristic of an Aloe Vera in charging a capacitor for a long duration

**Fig 14:**
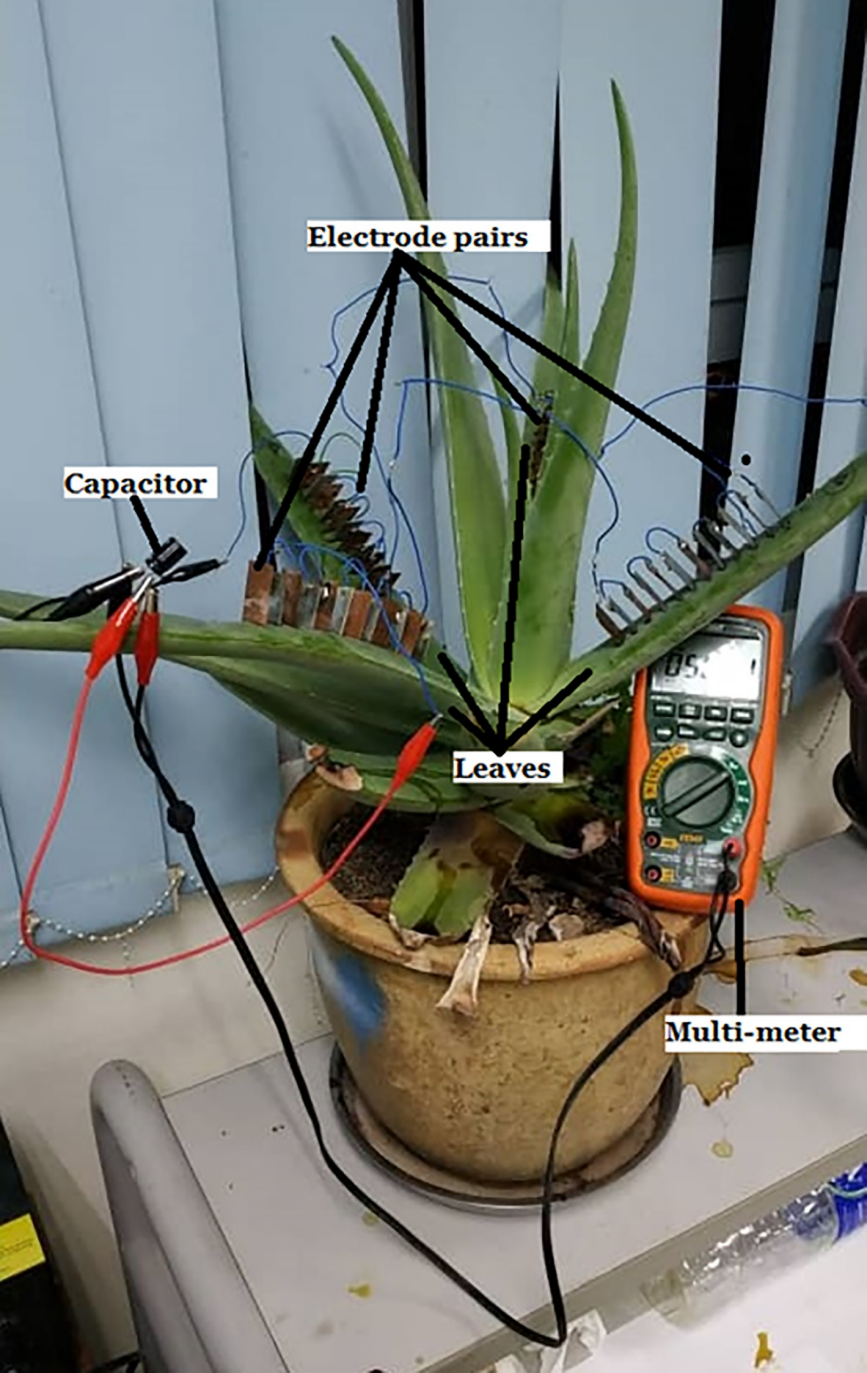
Experiment setup for charging a capacitor with series a connection of leaves, which passes through the stem of Aloe Vera.

**Fig 15:**
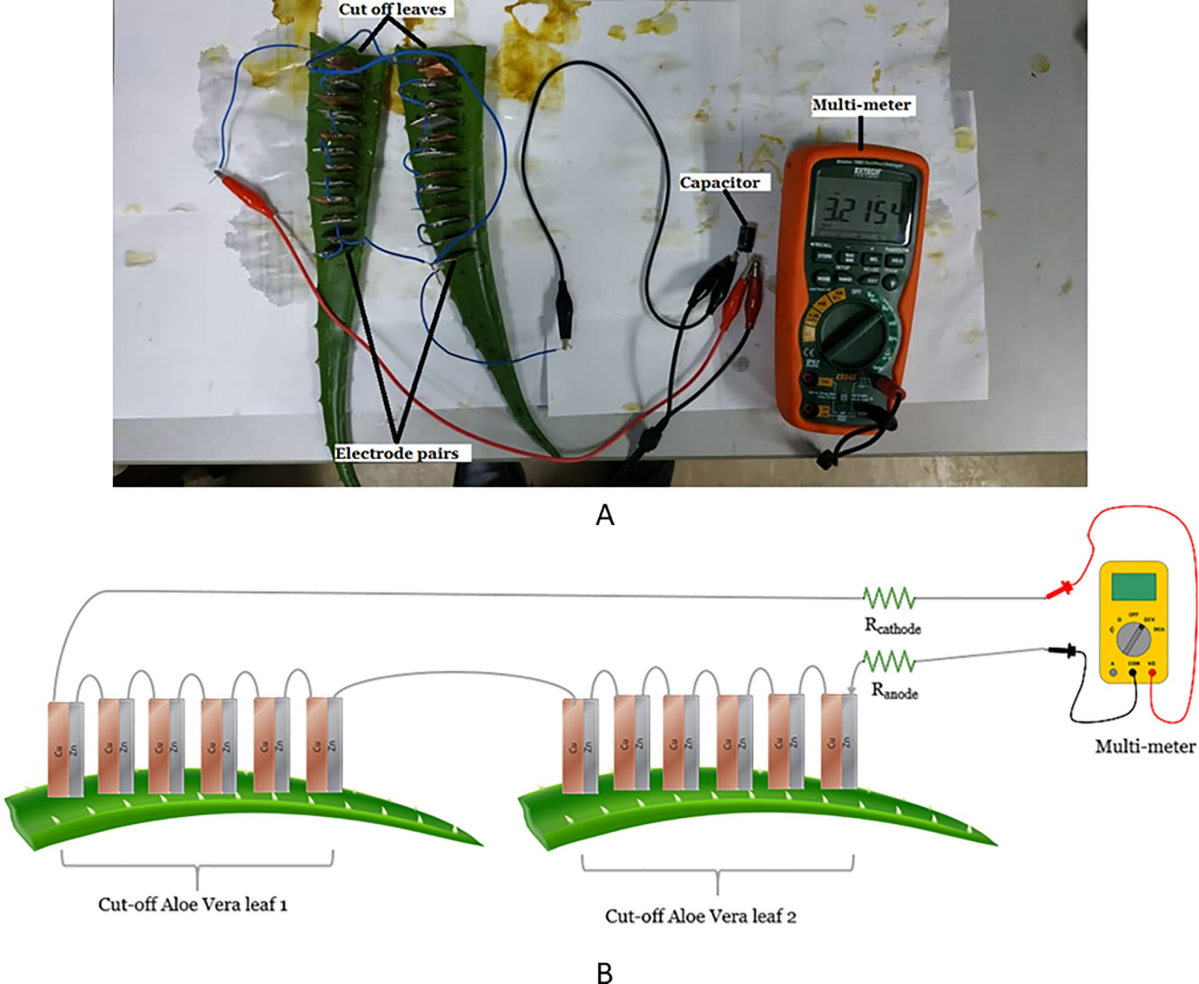
Experiment setup for charging a capacitor with cut off leaves disconnected from the Aloe Vera. Example of setup arrangement (A) and simple schematic of the setup (B).

This experiment is performed to determine the voltage characteristic of the PBC system in charging an output capacitance as a storage reservoir for a long duration of time. The duration is fixed to 3 days. It is carried out in two different scenarios as shown in Fig 14 (similar to Fig 13(B) setup) as well as Fig 15 respectively

(i) A series connection of leaves, which passes through the stem of Aloe Vera.

(ii) A series connection of cut off leaves disconnected from the Aloe Vera.

Two leaves are used. Six electrodes pair are embedded in each leaf. The electrodes are connected via a series connection. The output capacitor selected is a 16V, 1000 uF capacity.

## Results and discussion

### Investigation of the types of electrodes

Table 2, summarizes the results of each electrode pair combination used to harvest voltage and current from the plant. The result shows that the best combination for harvesting the highest amount of voltage and current from the plant is copper and zinc electrodes. This is because copper and zinc combination are more reactive in the electrochemical series compare to other combinations of electrodes used in this experiment. It causes both electrodes to generate a higher amount of electricity when embedded into the Aloe Vera leaf. Oxidization, which occurs in zinc electrode, causes the Zn atom to change into Zn2+ ion and releases electrons from the zinc electrode (Zn(s) → Zn2+ (aq) + 2e-) which flows through the external wire to the load (multi-meter) and later towards the copper electrode. Reduction takes place at the copper electrode. The Aloe Vera inner semi-solid fleshy gel acts as the electrolyte.

**Table 2:** Voltage and current from different types of electrode pairs.

Number	Electrode types		Mean voltage measured without load (V)	Mean current measured without load (uA)
	Cathode(+)	Anode(-)		
1	Copper	Nickel	0.156	9
2	Aluminum	Zinc	0.406	76
3	Nickel	Aluminum	0.420	79
4	Copper	Aluminum	0.624	117
5	Nickel	Zinc	0.837	159
6	Copper	Zinc	0.9851	205

The direction of the electron flow is determined by the ease of oxidization level between two electrodes. As zinc is more reactive than copper and it has a stronger tendency to lose electrons, hence this causes the electron to flow from zinc electrode to copper electrode. The whole system, which operates similar to a voltaic cell, makes the zinc electrode as the anode, the negative terminal and the copper as the cathode, the positive terminal. This gives the current, which is holes to flow from copper (cathode) to zinc (anode).

Table 3 gives the comparison of harvested voltage and current from various plants available in the literature with our Aloe Vera plant. We have compared the results with the succulent plant and non-succulent plants.

**Table 3:** Comparison of voltage and current harvested between different types of living plants with Aloe Vera.

Type of plants	Name of plant	Voltage, V	Current, uA	Method of harvesting
Non-succulent	Bigleaf maple tree	0.05–0.23	0.5–2.3	Using two steel nails, one inserted into the tree trunk and another inserted on the soil 30cm away from the tree [27].
Non-succulent	Pachira tree	0.80	3.0	Using galvanized iron nails inserted into the tree trunk and a stainless steel electrode planted in the soil nearby [28].
Non-succulent	Poplar tree	0.897–0.932	47.37–49.55	Using two copper electrodes, one inserted into the tree trunk and another inserted on the tamped soil 30cm away from the tree [29].
Non-succulent	Avocado plant	0.52–0.67	1.54–2.08	Using a zinc anode alloy in a combination with copper cathode as a pair of electrodes inserted into the tree trunk [30].
Non-succulent	Pulai tree	0.80	Non-available	Using copper-zinc electrodes inserted into the tree trunk [24].
Non-succulent	Banana tree	0.913	Non-available	Using copper-zinc electrodes inserted into the tree trunk [24].
Succulent	Aloe Vera	0.9851–0.988	187–205	Using copper-zinc electrodes immersed into the succulent leaf of the plant as in this experiment.

From Table 3, it is observed that the Aloe Vera leaf, which is inserted with a single pair of copper-zinc electrodes, is able to harvest approximately 0.9851 V to 0.988 V and 187uA to 205uA current, which is higher in magnitude compared to other plants.

### Investigation on the distance between electrodes

Table 4 shows the experimental results of harvested voltage and current by varying the distance between the copper and zinc electrode pair. From the result, it is observed that the smaller the distance between the copper and zinc electrode pair embedded into the Aloe Vera leaf, the higher the amount of voltage and current can be harvested from the plant. This can be explained based on electrochemistry theory where the maximum electron transfer only happens when electrodes are at a very close distance. Thus, by moving the electrodes closer, the resistance decrease and this enables the current to increase, hence allowing an easier transfer of electrons.

**Table 4:** Voltage and current measured by varying the distance between an electrodes pair.

Electrodes distance, cm	Mean voltage measured, V	Mean current measured, uA	Position on leaf
1	0.988	187	Start of leaf from the stem
2	0.987	187	
3	0.984	186	Middle of the leaf.
4	0.979	185	
5	0.973	184	
6	0.956	182	
7	0.951	180	
8	0.940	179	
9	0.935	178	
10	0.925	175	
11	0.923	175	End of the leaf towards the tip.
12	0.917	173	

### Investigation on the different parts of an Aloe Vera to embed electrodes

**Fig 16:**
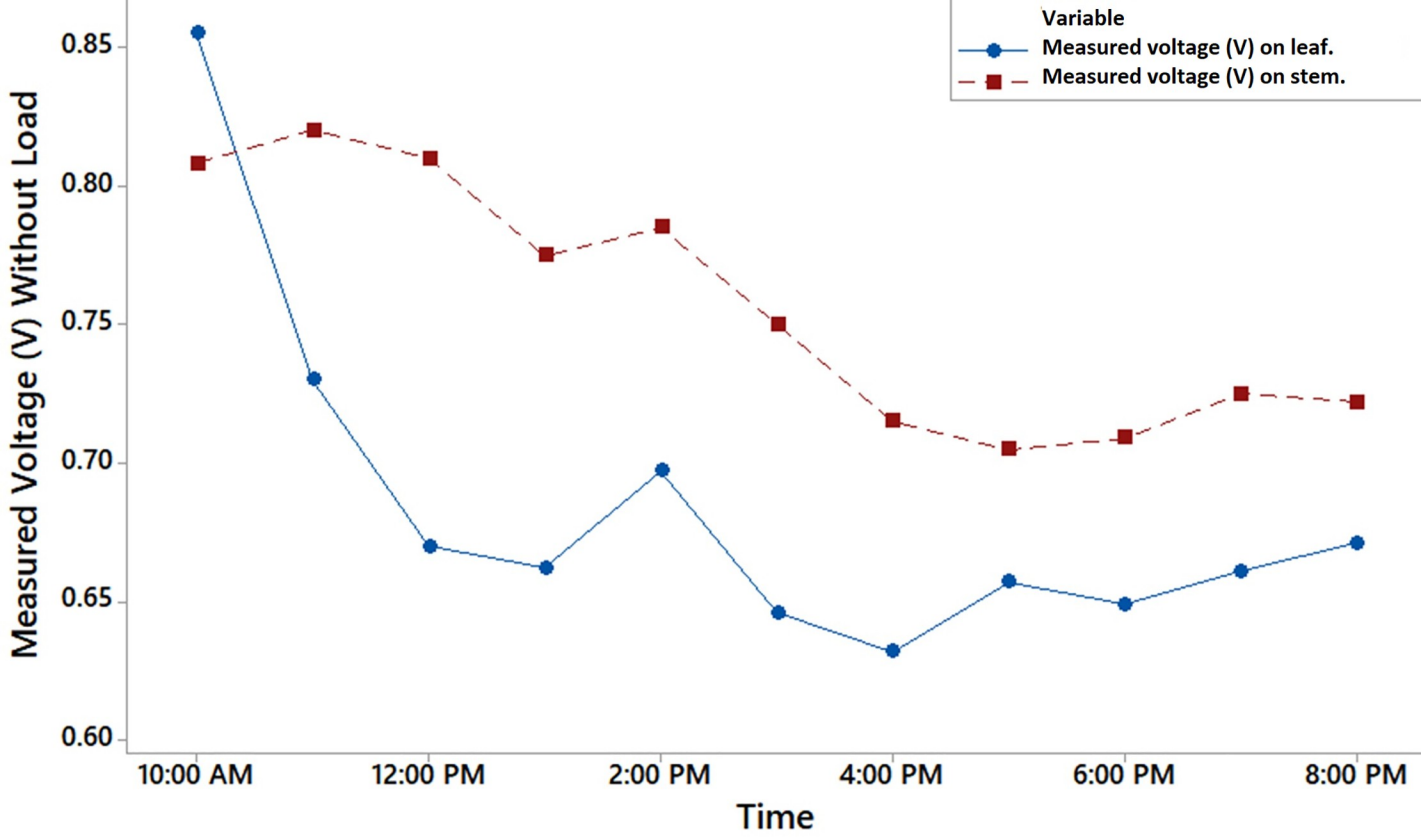
Comparison of voltage harvested from Aloe Vera leave and stem.

Fig 16 shows the variation between the measured voltage at the leaf and at the stem of the plant against the time. It is observed that in the morning the voltage harvested from the Aloe Vera leaf is higher than the voltage harvested from the stem. However, as the time progress, the voltage harvested from the stem is higher than the voltage harvested from the leaf at about 9.5%. This result suggests that the excretion of the fluid sap out from the inner flesh gel of the leaf, which acts as an electrolyte at the position where the electrode is embedded, reduces the volume of the conducting medium between the electrodes as time progress. As the amount of the inner flesh gel reduces, the value of the harvested voltage drops. There is no excretion of sap observed on the stem of Aloe Vera where the electrode pair is embedded. Although the result shows that the voltage harvested from the stem is higher than the leaf, harvesting the energy from the leaf is more preferable because an Aloe Vera has multiple leaves but only a single stem.

### Investigation on the effect of the number of electrodes used

Table 5 summarizes the magnitude of voltage and current harvested from an Aloe Vera leaf when a different number of electrode pairs are used. The maximum number of 6 electrode pairs are used due to the limitation of the space available on a single leaf. Experimental results suggest that by connecting the higher number of electrode pairs in series, a larger amount of power can be harvested. In addition, it shows that the voltage output is proportional to the number of electrodes, which is in accordance with the Kirchhoff voltage law since, each electrode pair acts as a cell, which contributes as a voltage source and connecting them in series increases the total output voltage. On the other hand, as more electrodes are connected in series, the current decreases due to the introduction of higher internal resistance among the connected electrodes. However, it is also observed that the output power increases as the number of electrodes increases. The maximum harvested power is 496.8uW by using 6 pairs of electrodes in a single leaf.

**Table 5:** The magnitude of voltage and current harvested.

Electrode pairs	Voltage measured, (V)	Current measured, (uA)	Power measured, (uW)
1	0.98	205	200.9
2	1.5	186	279
3	1.86	175	325.5
4	2.19	165	361.35
5	2.71	150	406.5
6	3.6	138	496.8

### Investigation on the effect of series and parallel connection for electrodes

Table 6 tabulates the comparison of voltage and current harvested when 4 leaves labeled as leaf 1 to leaf 4 are connected in series and parallel. Under no load condition, the series connection among the leaves produces a higher amount of voltage when more leaves are connected in series. However, there are no significant changes in the harvested current. On the other hand, the parallel connection among the leaves produces a higher amount of current when more leaves are connected in parallel. However, it does not give a distinctive increment in voltage. Therefore, a combination of series and parallel connection among the leaves can be utilized to harvest either higher voltage or current. It is worth to note that 908.39uW can be harvested when 4 leaves are connected in series while 1111.55uW can be harvested from leaves when they are connected in parallel.

**Table 6:** Comparison of voltage and current from series and parallel connection between Aloe Vera leaves.

Connection method	Leaf Number	Voltage (v)	Current (uA)	Power (uW)
Individual leaf	1	2.96	220	651.2
	2	3.45	130	448.5
	3	4.24	128	542.7
	4	3.79	166	629.1
The series connection between leaves	1 and 2	5.45	134	730.3
	1,2 and 3	5.89	144	848.16
	1,2,3 and 4	6.83	133	908.39
The parallel connection between leaves	1 and 2	2.35	295	693.25
	1,2 and 3	2.48	385	954.8
	1,2,3 and 4	2.35	473	1111.55

### Investigation on the variation of series connection for leaves

**Fig 17:**
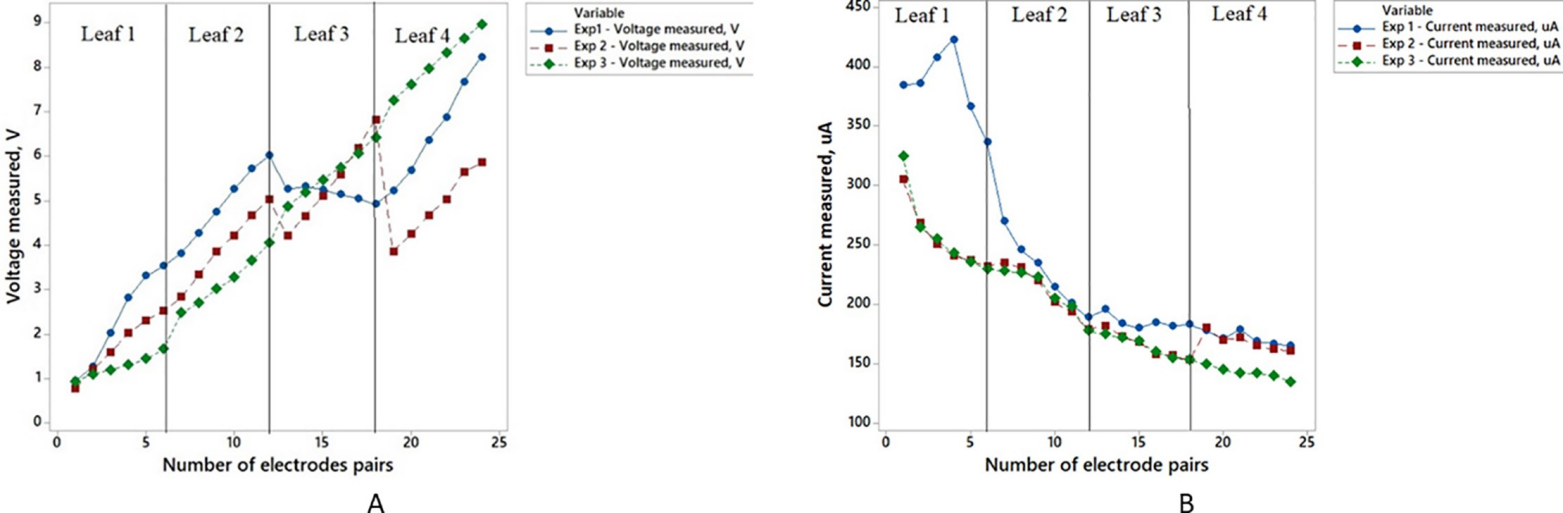
Comparison of electrical output for Exp1-3. Comparison in terms of output voltage (A) and output current (B).

The results for this investigation are shown in Fig 17 for the three different types of series connection setup:

- Series connection among leaves which is passing through the stem of the Aloe Vera (denoted as Exp 1).
- Series connection among the leaves which by-pass the stem of Aloe Vera (denoted as Exp 2).
- Series connection among cut off leaves which are not connected to the stem of Aloe Vera (denoted as Exp 3).

It is observed that the series connection among leaves which is passing through the stem of the Aloe Vera (referred as Exp 1) harvest much higher voltage and current compared to the series connection among the leaves which by-pass the stem of Aloe Vera (referred as Exp 2). However, it is observed that in Exp 1, the magnitude of voltage increases linearly from Leaf 1 to Leaf 2 connection, but it falls when it is connected from Leaf 2 to Leaf 3. The same phenomenon also occurs in Exp 2 where the connection between Leaf 2 to 3 and Leaf 3 to 4 produce a significant drop in voltages. It is believed that the internal connection of each leaf towards the plant stem causes the voltage drop on the electrode pairs connection when one leaf is connected to another. To prove this inference, a series connection among cut off leaves, disconnected from the stem of Aloe Vera (referred to as Exp 3) is carried out. It shows that the voltage increases linearly when the cut off leaves are connected in series. Thus, by comparing the three results, it is concluded that the significant drop of voltage between Leaf 2 to 3 and Leaf 3 to 4 in Exp 1 and Exp 2 are due to the internal connection between leaves to stem of Aloe Vera. On the other hand, the currents harvested from each Exp 1–3 decrease as the number of electrodes increases. However, it is observed that Exp 1 can harvest the highest amount of current from the plant.

Hence, this experiment proves that the series connection between leaves which is passing through the stem of the Aloe Vera with electrode pairs embedded into them (Exp 1) is preferable as it can produce a higher voltage and current compared to Exp 2 with series connection by-passing the stem.

### Investigation on charging capability of Aloe Vera towards different capacitors

**Fig 18:**
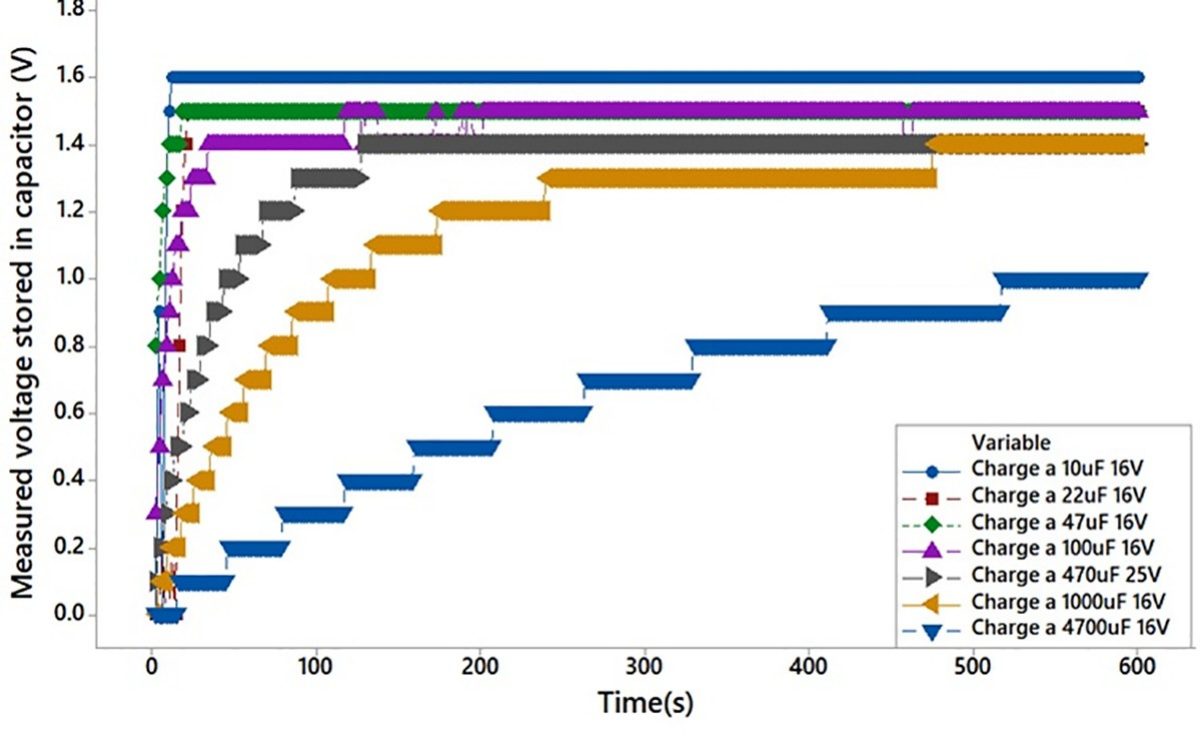
Tabulation of voltage measured from a range of capacitors charge by the PBC at a fix duration.

Fig 18 shows the feasibility of the PBC system to charge a capacitor and the amount of voltage stored in various capacitors for a fix time duration of 600 seconds. The experimental finding suggests that the PBC system is capable to charge all the capacitors used in this experiment. It is observed that a 10 uF, 16 V capacitor can be charged up to 1.6 V in approximately 10 seconds while a 4700 uF, 16 V capacitor can store only 1 V in 600 seconds due to large time constant which is mathematically expressed in (1);

τ=RC (1)

Hence, it is observed that the electric energy generated from Aloe Vera can be used to charge a capacitor. The capacitance value of the capacitor must be chosen accurately to fit the requirement of operating an output load in terms of its power efficiency and operating cycle.

### Investigation on the characteristic of an Aloe Vera in charging a capacitor for a long duration

**Fig 19:**
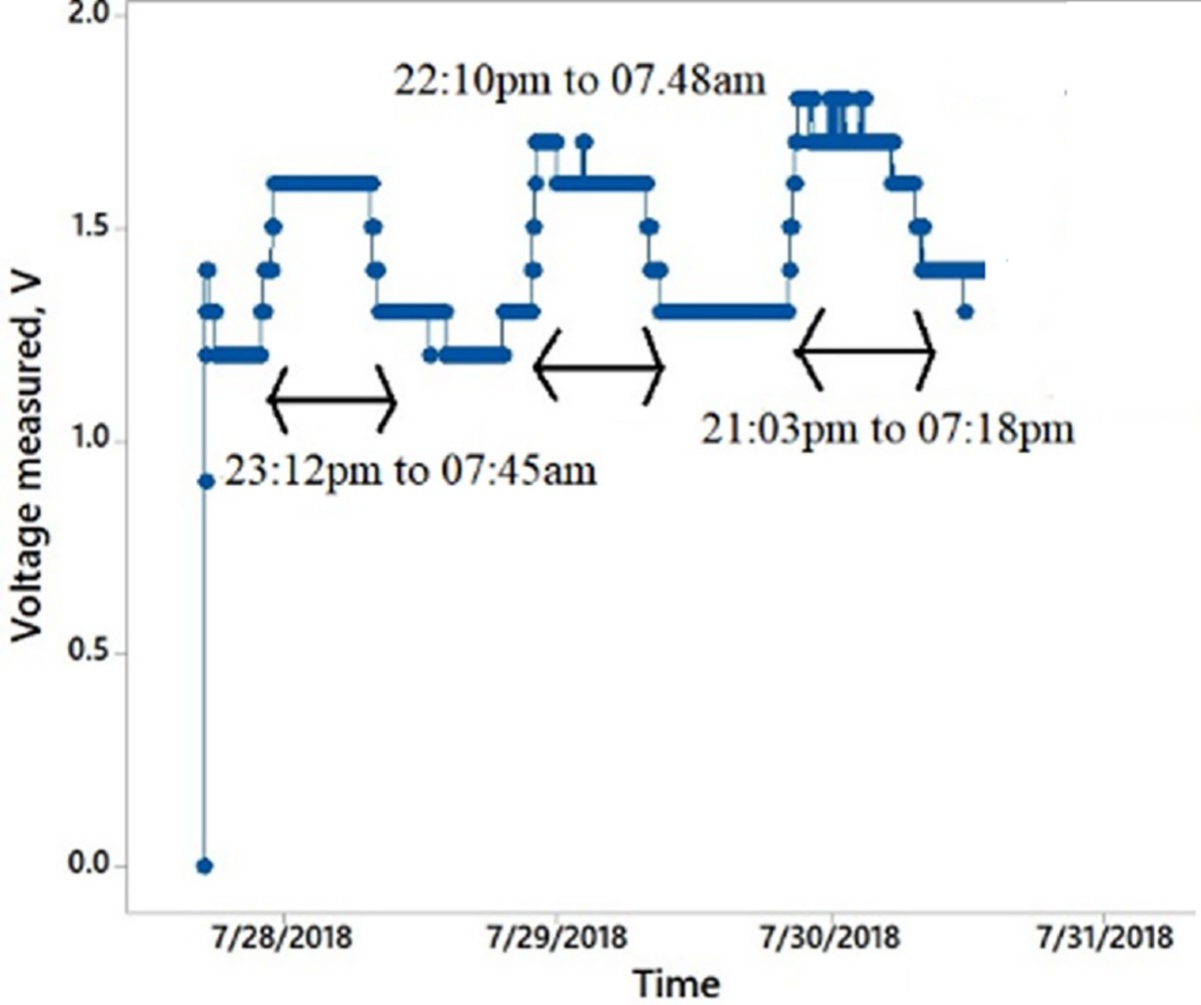
Voltage value of a capacitor, which is charged by a series connection between two leaves which passing through the stem of Aloe Vera over a period of 3 days.

**Fig 20:**
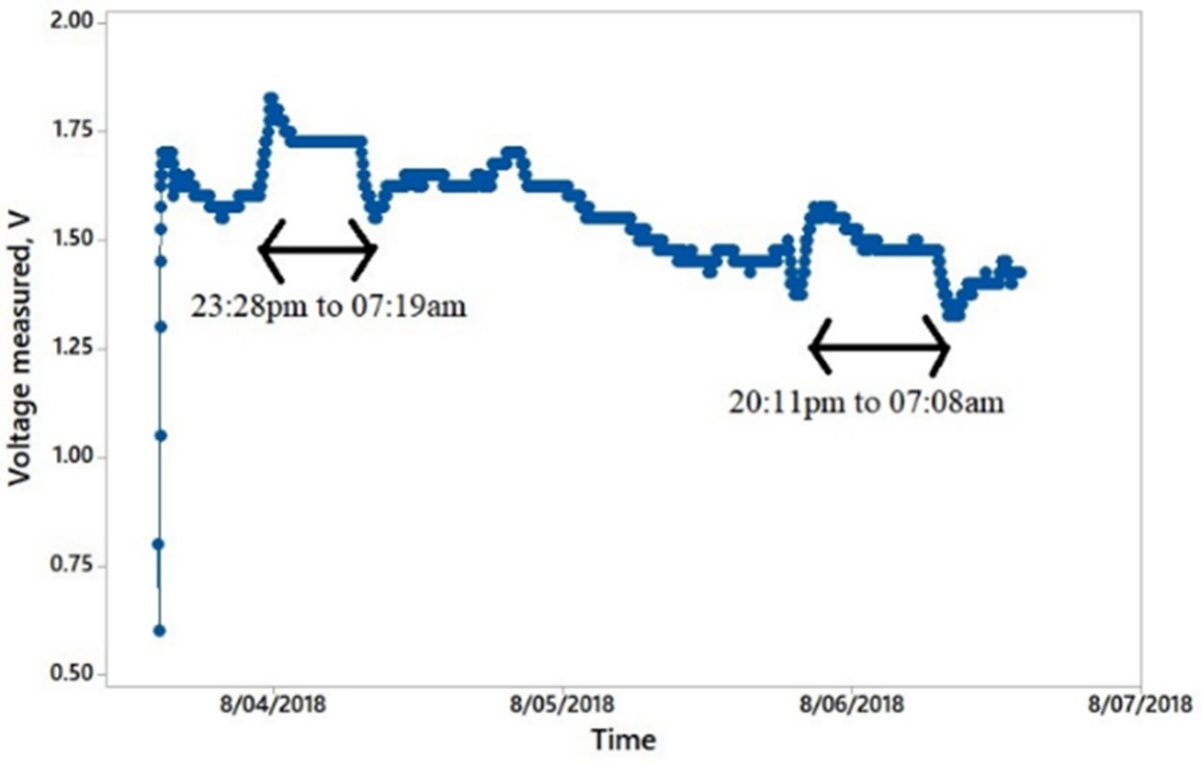
Voltage value of a capacitor, which is charged by a series connection of cut off leaves disconnected from the plant over a period of 3 days.

Figs 19 and 20 show the experimental results of charging a capacitor by using series connection between two leaves which passing through the stem of Aloe Vera and the later on the series connection of cut off leaves disconnected from the plant for a period of 3 days.

In Fig 19, it is observed that the charging voltage from the setup shows a continuous charging of the output capacitor with increment in the magnitude of the charging voltage periodically. A ripple exists in the charging behavior. The charging voltage towards the capacitor increases and decreases approximately during the 8.00 pm to 8.00 am every day. This shows that the plant generates more energy at night due to its respiration process. It is also found that the plant can detect a light stimulus and use the stimulus as a timing process for its photosynthesis and respiration cycle. This ability is termed as photoperiodism. On the other hand, Fig 20 shows a decreasing magnitude of voltage over time. It also shows that the characteristic of higher charging capability at night approximately between 8.00 pm to 8.00 am but with less frequent repetition of the event because the leaves, which are disconnected, from the plant have lesser photoperiodism ability. By comparing both results, it is proven that harvesting the energy from the leaves connected to the plant is a feasible method as it provides a continuous increment in charging voltage for a capacitor. Disconnected leaves from the plant provide a decreasing charging voltage for the capacitor. The plant is capable of charging a capacitor continuously during the day and night as it proceeds with its photosynthesis and respiration cycles. Hence, the charge stored in the capacitor can be used to power up an ultra-low power consumption device.

## Conclusion

It is concluded that the Aloe Vera plant can generate electrical energy, which can be potentially useful to power up ultra-low power consumption devices. As compared to other living plants used in other researches to harvest energy, Aloe Vera has been observed to generate the highest magnitude of voltage and current. This energy can be stored in a capacitor. From the results of the experiments, it is observed that copper as the cathode electrode and zinc as the anode electrode is the best combination to generate maximum voltage and current. The shorter the distance between two electrodes embedded in the plant, the higher the voltage and current harvested. We have also seen that the harvested voltage or current can be increased by connecting a higher number of electrode pairs in series or in parallel. A series connection of the Aloe Vera leaves, which are inserted with copper-zinc electrodes, can generate a higher voltage. On the other hand, a parallel connection of the Aloe Vera leaves, which are inserted with copper-zinc electrodes, can generate higher current. Hence, a combination of series and parallel connection between the Aloe Vera leaves can be used to generate the optimum amount of voltage and current to power a desired low power consumption device. In addition, it is also observed that if the connection of electrode pairs in series passes through the plant stem, then higher voltage and current can be generated. Moreover, the energy harvested from the Aloe Vera plant is capable of charging the capacitor in a continuous periodical pattern in day and night. The charging rate of the capacitor is higher during night time.

Hence, from this research, it is proven that electrical energy can be tapped from Aloe Vera leaves and it can be optimized to meet the desired voltage and current value via various experiment setups. This green energy, which can be stored in a capacitor, can be potentially used to power up ultra-low power devices such as remote sensors where energy is scarce in remote areas in future works.
